# Case report: Successful use of ruxolitinib to treat interstitial pneumonia as an unusual primary presentation in primary myelofibrosis—two birds with one stone

**DOI:** 10.3389/fonc.2024.1475036

**Published:** 2024-11-26

**Authors:** Pingping Xiao, Zhigao Dong, Qingqing Wang, Junnan Su, Yongquan Chen, Yiwan Lin

**Affiliations:** Department of Hematology and Rheumatology, The Second Affiliated Hospital of Xiamen Medical College, Xiamen, China

**Keywords:** interstitial pneumonia, Janus kinase 2, primary myelofibrosis, ruxolitinib, ten-eleven translocation 2

## Abstract

**Background:**

Interstitial lung disease (ILD) is a rare clinical presentation of primary myelofibrosis (PMF).

**Case presentation:**

We report a case of ILD as the main manifestation on admission. A 58-year-old man was diagnosed with PMF owing to worsening anemia following treatment failure for conventional interstitial pneumonia.

**Results:**

Anemia and interstitial pneumonia both significantly improved following treatment with a Janus kinase 2 gene inhibitor. In this report, we discuss the possible mechanisms underlying PMF complicated with ILD.

## 1 Introduction

Primary myelofibrosis (PMF) is a hematologic malignancy characterized by clonal expansion of one or more myeloid lineages, resulting in excessive hematopoietic cell production (1). Interstitial lung disease (ILD), with its rapid progression and poor prognosis, causes respiratory failure and poses a serious threat to life and health (2). Here, we report a case of PMF with ILD as the main manifestation. Our patient was diagnosed with PMF following conventional interstitial pneumonia treatment. Following treatment with a Janus kinase 2 (*JAK2*) gene inhibitor, his blood test results and his interstitial pneumonia significantly improved.

## 2 Case description

A 58-year-old man presented at our hospital with a 1-year history of cough that had worsened over the previous week. Physical examination findings were as follows: heart rate, 113 beats/min; respiratory rate, 28 breaths/min; blood pressure, 126/72 mmHg; and oxygen saturation (pulse oximetry), 92%. No clinical symptoms of enlarged superficial lymph nodes or liver or spleen symptoms were reported. He had no respiratory-relevant personal or familial medical history. His laboratory examination results were as follows: hemoglobin (Hb), 81.0 g/L [normal range (NR), 130–175 g/L]; white blood cell count, 16.96 × 10^9^/L (NR, 3.50–9.50 × 10^9^/L); platelet count, 165 × 10^9^/L (NR, 125–325 × 10^9^/L); reticulocyte count, 131.98 × 10^9^/L (NR, 20–200 × 10^9^/L); and reticulocyte ratio, 1.12% (NR, 0.3–3.0). The erythrocyte sedimentation rate was increased at 40 (NR, 0–15) mm/h. His serum glutamic oxaloacetic transaminase, serum glutamic pyruvic transaminase, serum γ-glutamyl transpeptidase, serum lactate dehydrogenase, and serum alkaline phosphatase levels were 16.90 IU/L (NR, 15.0–40.0 IU/L), 12.10 IU/L (NR, 9.0–50.0 IU/L), 78 IU/L (NR, 10.0–60.0 IU/L), 307.89 IU/L (NR, 100.0–240.0 IU/L), and 84 IU/L (NR, 45–125 IU/L), respectively. His creatine kinase and creatine kinase isoenzyme levels were within the NR. A sputum culture was negative; however, a sputum smear was positive for *Mycobacterium tuberculosis*. Serum tests for nine respiratory pathogens (Q fever rickettsial disease, influenza A virus, influenza B virus, respiratory syncytial virus, parainfluenza virus, *Mycoplasma pneumoniae*, *Chlamydia pneumoniae*, adenovirus, and *Legionella pneumophila*) yielded negative results. Polymerase chain reaction tests for coronavirus disease yielded negative results on three separate occasions, and his thyroid function test results were normal. Epstein–Barr virus DNA and cytomegalovirus (CMV) DNA levels were low (<4.0 × 10^2^ copies/mL). Other test results were within the NR, as follows: arterial blood gas, pH 7.38 (NR, 7.35–7.45); partial pressure of oxygen, 59.40 (NR, 80–100) mmHg; partial pressure of carbon dioxide, 36.40 (NR, 35–48 mmHg) mmHg; and blood lactate level, 2.22 (0.5–2.0) mmol/L. Immunoglobulin and complement C4 levels were normal; however, the complement C3 level was reduced at 0.25 (NR, 0.9–1.8) g/L. Antinuclear antibodies, antiphospholipid antibodies, anti-extractable nuclear antibodies, anticyclic citrullinated peptide tests, myositis-specific autoantibodies, antineutrophil cytoplasmic antibodies, and rheumatoid factor were negative.

**Figure 1:**
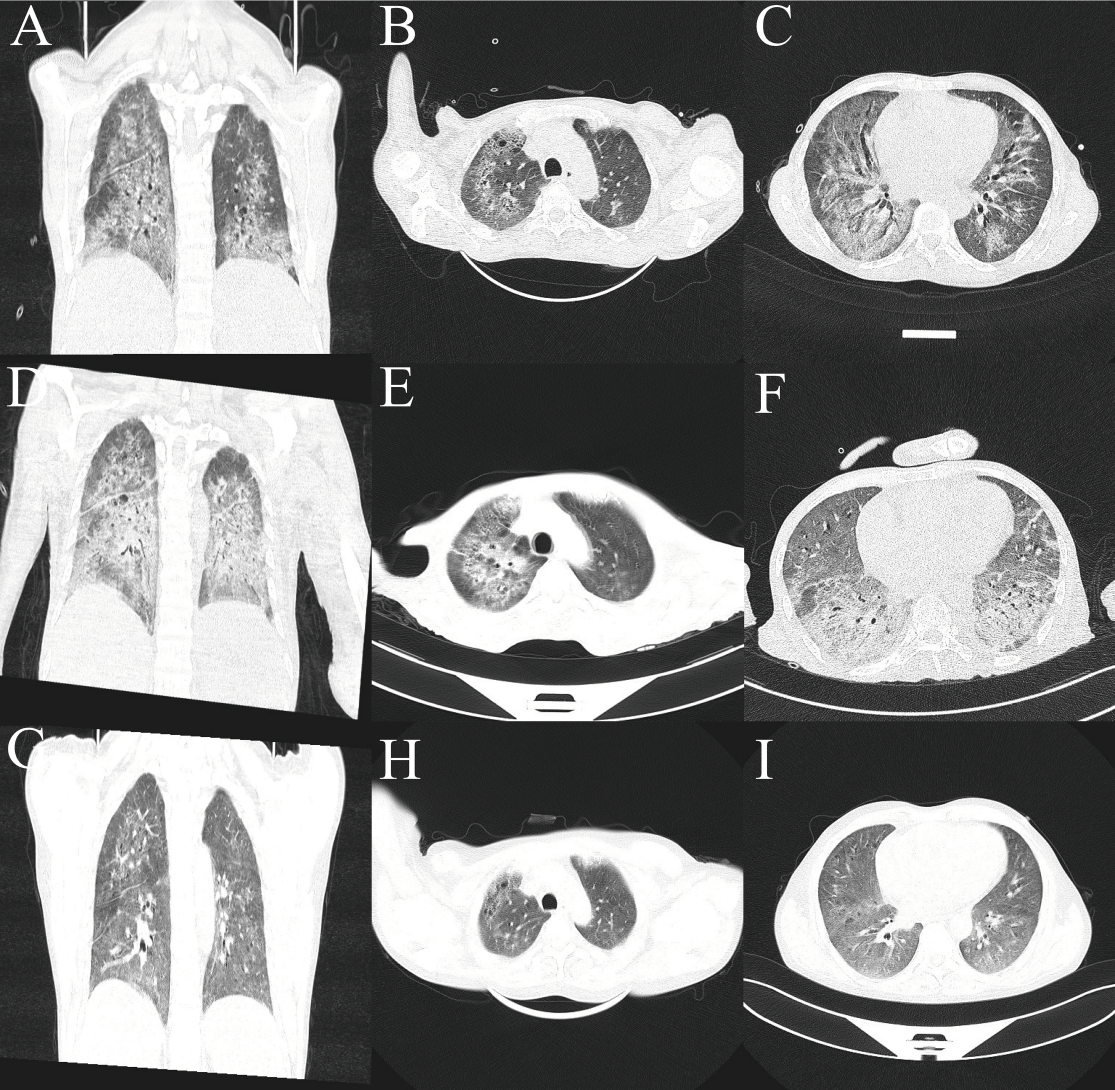
Lung computed tomography. **(A–C)** At admission. **(D–F)** Axial view after methylprednisolone and cyclophosphamide therapy. **(G–I)** After treatment using ruxolitinib, a *JAK2* inhibitor.

**Figure 2:**
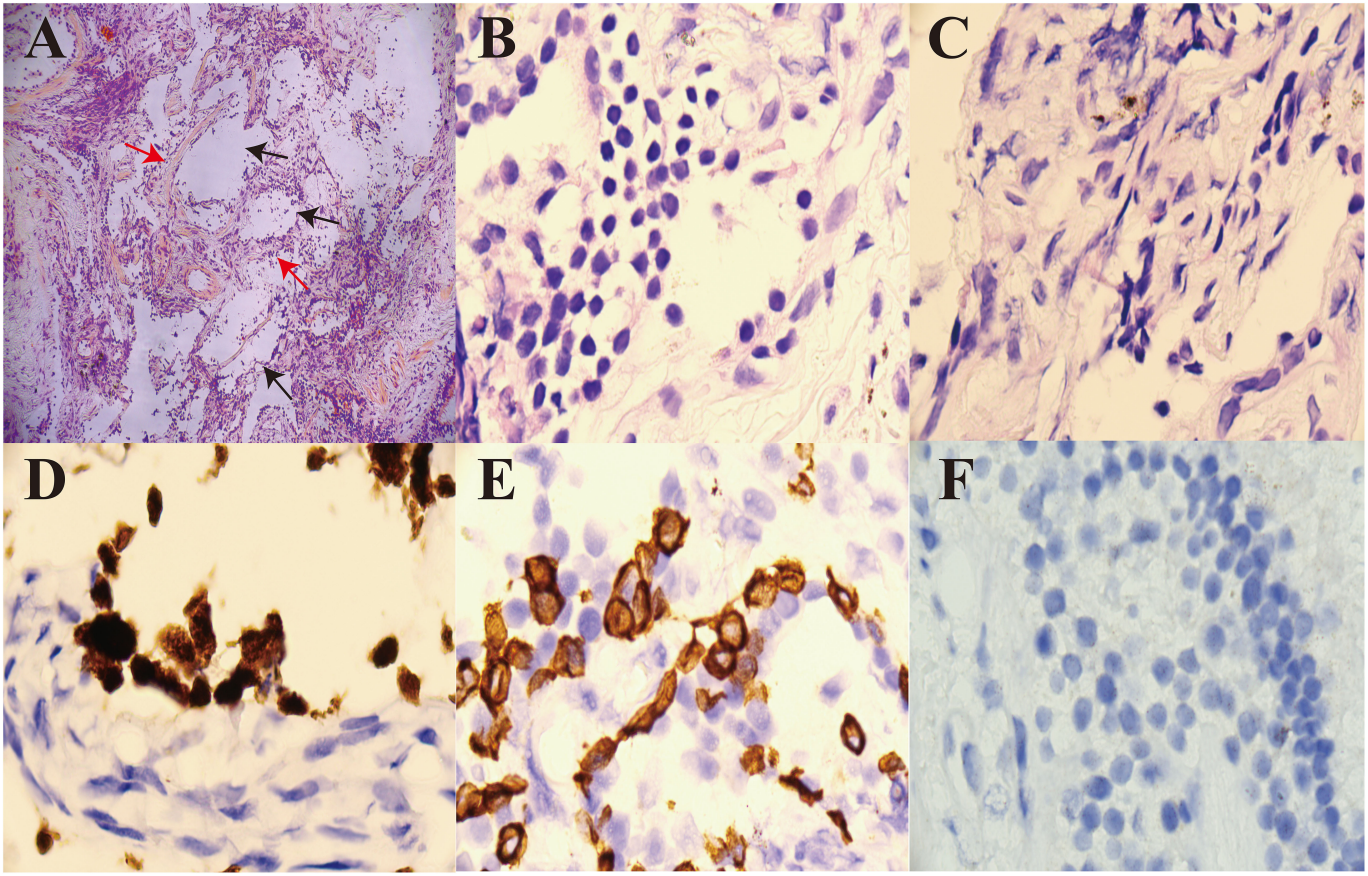
Pathological analysis of a percutaneous dorsal segment of the right lower lobe lung biopsy. **(A)** H&E staining shows dilated alveolar spaces (black arrow, ×100) and fibroblastic cell proliferation in the interstitium (red arrow, ×100). **(B)** H&E staining shows that the infiltrating cells were lymphocyte cells (×1,000). **(C)** H&E staining shows fibroblastic proliferation fibrosis with collagen (×1,000). **(D)** CK5/6 positive (×1,000). **(E)** TTF-1 positive (×1,000). **(F)** Acid-fast staining did not detect any acid-fast bacilli (×1,000). H&E, hematoxylin and eosin.

On admission, high-resolution computed tomography (CT) of the chest showed diffuse ground-glass opacities in the lungs and interstitial pneumonitis (**Figures 1A–C**). Metagenomic sequencing of the bronchoalveolar lavage fluid sample revealed no viral RNA, viral DNA, bacteria, fungi, mycoplasmas, chlamydia, or parasites. Pathology results in relation to bronchoalveolar lavage fluid showed the following: the color was light red, transparency was slightly turbid, 77% of the total cells were neutrophil cells, lymphocytes comprised 8%, macrophages comprised 9%, and ciliated epithelial cells comprised 6%, with no evidence of infection or malignant cells. A punch biopsy of the lung lesion was also performed, with results showing dilated alveolar spaces, alveolar epithelial hyperplasia, widened alveolar septum, fibroblastic proliferation fibrosis with collagen, and lymphocyte cells as infiltrating cells. With bronchiolar metaplasia, morphological characteristics conformed to interstitial pneumonia (**Figure 2**).

**Figure 3:**
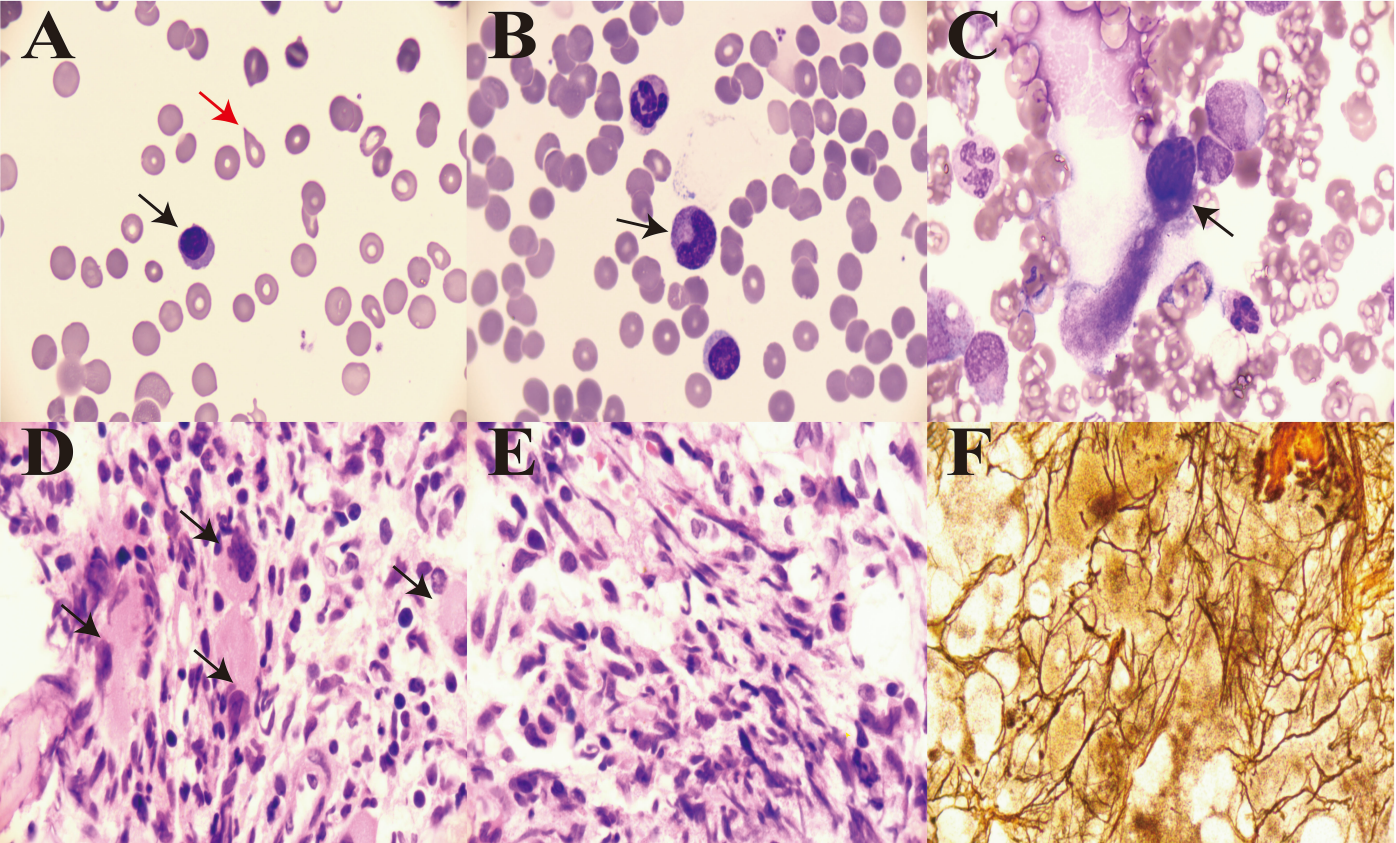
Bone morphology and pathology from a bone marrow biopsy. **(A)** Teardrop poikilocytes (red arrow) and occasional late erythroblasts in peripheral blood (×1,000, black arrow). **(B)** Late myelocytes in peripheral blood (×1,000, black arrow). **(C)** Abnormal megakaryocytes (×1,000, black arrow). **(D)** Cluster distribution of myeloblasts in marrow (×1,000, black arrow). **(E)** Diffuse increase in reticular fibers in marrow (×1,000). **(F)** Reticulin stain showing widespread positivity (×1,000).

The patient was diagnosed with ILD and received methylprednisolone (500 mg/day for 3 days and tapering subsequently) and cyclophosphamide (0.4 g per week) combined with gamma globulin (0.4 g/kg per day for 5 days). However, the patient’s wheezing and cough did not improve, and a lung CT scan also indicated there was no improvement (**Figures 1D–F**). During treatment, his anemia worsened, and his anemia-related examination results were as follows: serum ferritin, 328.50 (NR, 11.0–306.8) ng/mL; serum iron, 32.27 (NR, 11–30) µmol/L; total iron-binding capacity, 38.27 (NR, 45–75) µmol/L; transferrin saturation, 84.32%; serum B12, 574.96 (NR, 180–914) pmol/L; serum folate, 18.76 (NR, 11.8–50.0) nmol/L; and reticulocyte percentage, 2.72% (NR, 0.3–3.0%). Direct and indirect antiglobulin test results were negative. A bone marrow puncture was performed. A peripheral blood smear revealed the presence of teardrop poikilocytes, occasional late erythroblasts, and late myelocytes (**Figures 3A, B**). Bone marrow morphology results revealed myeloid proliferation, megakaryocytic hyperplasia, and abnormal megakaryocytes (**Figure 3C**). Flow cytometry results indicated no developmental abnormalities in the bone marrow. Pathological findings from a bone marrow biopsy revealed the presence of myeloproliferative disease, namely, PMF. Immunohistology analysis findings were as follows: negative for CD2, CD5, CD20, PAX5, CD30, cyclin D1, CK, EMA, BCL2, Bcl6, C-myc, and EBER; and positive for CD10 and MUM1, Ki-67 (+50%–60%), and reticular fibers (2+) (**Figures 3D–F**). Bone marrow metaphase cytogenetics revealed a 46,XY karyotype. Positive *JAK2/V617F* and *TET2* gene mutations were also identified, whereas the *BCR-ABL* mutation was absent.

Following the diagnosis of PMF, we administered a *JAK2* inhibitor (ruxolitinib, 20 mg twice daily). One week later, the size of the lung lesions had improved significantly (**Figures 1G–I**). His Hb levels gradually increased, and interleukin (IL)-6 levels decreased (**Table 1**). His condition improved over 2 weeks, and he was discharged. At 12-month follow-up, the patient could engage in physical activities and showed no recurrence.

**Table 1:** Indices before and after different treatments.

Index	WBC	Hb	PLT	CRP	IL-6	Ferritin
Normal range	3.5–9.5 ×10^9^/L	130–175 g/L	125–325 ×10^9^/L	0–10 mg/L	0–10.3 pg/mL	11.0–306.8 ng/mL
At admission	16.96	81	165	1.76	406	328.50
Following methylprednisolone and cyclophosphamide therapy	28.24	78	92	10.45	523	423.60
Following *JAK2* inhibitor treatment	16.54	121	268	5	3.32	203.20

CRP, C-reactive protein; Hb, hemoglobin; IL-6, interleukin-6; PLT, platelet; WBC, white blood cell.

## 3 Discussion

This patient presented to the hospital with interstitial pneumonia as the main manifestation on admission. He received glucocorticoids, cyclophosphamide, anti-fibrotic agents, gamma globulin, and anti-infective therapy, but his condition did not improve and became progressively aggravated. Following a diagnosis of myelofibrosis and treatment with *JAK2* inhibitors, his ILD improved significantly. This case report focused on the ILD, which is known to be mostly caused by secondary immune system disease, and its pathogenesis results from immune-related factors that cause lung lesions. Treatment typically targets the primary disease causing the ILD (3, 4). Our patient was diagnosed with ILD based on his clinical presentation and pathological lung lesion biopsy findings, as the primary cause was unclear. A diagnosis of ILD was made based on the etiological examination findings shown in **Figure 2**.

Owing to worsening anemia during treatment, a bone marrow aspiration and a pathological biopsy were performed, and he was diagnosed with PMF. Some cases of hematologic malignancy concomitant with ILD have previously been reported. Liu et al. reported the case of a male patient who had been diagnosed with myeloid neoplasm with secondary interstitial pneumonia (5). Li et al. reported on a patient who had been diagnosed with diffuse large B-cell lymphoma and ILD (6). Zou et al. reported on 321 cases of non-Hodgkin lymphoma presenting with ILD (7). However, reports concerning PMF present in patients with ILD are rare. The mechanism of action of *JAK2* inhibitors in treating ILD requires further discussion, and a clear understanding is needed as to why patients with myelofibrosis develop ILD. We first focused on the possible mechanisms related to ILD in patients with myelofibrosis, which is divided into primary and secondary myelofibrosis. Myelofibrosis presents with routine blood abnormalities, abdominal distension owing to spleen enlargement, weight loss, and fatigue, with primary lung disease lesions rarely observed (8). In addition to *JAK2/v617F* gene positivity in this patient, we also detected *TET2 Exon3* and *TET2 Exon11* gene mutations. *TET2* is essential for innate immune cell function. *TET2* is most profoundly expressed during murine macrophage differentiation compared with other *TET* genes. *TET2* is essential for controlling inflammation-inhibited IL-6 expression through recruiting histone deacetylase 2 (HDAC2). *TET2* has various mechanisms in neoplastic and immune diseases and downregulates the expression of inflammatory cytokines IL-6 and IL-1β through recruiting HDAC enzymes for histone deacetylation in innate myeloid cells and macrophages (9). *TET2-KO* macrophages and dendritic cells produce more IL-6. Loss of *TET2* in tumor-associated macrophages results in increased expression of inflammatory cytokines. Moreover, *TET2* regulates the differentiation of regulatory T cells and smooth muscle cells. *TET2* mutations counteract positive immune cell responses, including those of T cells and macrophages (10). The three primary IL-6 signaling pathways include classic signaling via the membrane-bound IL-6 receptor, trans-signaling using a soluble IL-6 receptor, and trans-presentation for antigen presentation (11). The relationship between IL-6 and ILD has previously been established (12). Stancil et al. identified the genetic or pharmacological targeting of the IL-6-related signaling pathway, leading to a reduction in fibrotic lung remodeling (13). From this perspective, the genetic mutations associated with PMF, especially *TET2*, may be one mechanism through which *TET2* mutation induces increased IL-6, leading to the immune-mediated inflammatory syndrome of pulmonary ILD.

A *JAK2* inhibitor (ruxolitinib) was initially used in the treatment of *JAK2/V617F*-positive myeloproliferative disorders. The treatment of ILD includes using *JAK* inhibitors such as tofacitinib (mostly *JAK1/3* inhibitors), which has achieved a certain clinical efficacy. *JAK2* inhibitors are considered to affect the release of IL-6 or inhibit its production, and a literature review was undertaken based on this consideration. Silva-Carmona reported that two patients with ILD were successfully treated using a *JAK2* inhibitor, namely, ruxolitinib (14). Lescoat et al. demonstrated that *JAK* inhibitors had anti-inflammatory properties, in part, based on results showing the activation state of pro-inflammatory macrophages and the downregulation of interferon β and IL-6 expression (15). Mima et al. reported that IL-6 increased inflammatory cytokine production and hypertrophic marker expression in primary mouse chondrocytes by activating the JAK2/STAT3 pathway (16). Li et al. generated *JAK2(V617F)*-expressing murine macrophages and reported that p-STAT3 levels were associated with increased IL-6 production (17). Cheng et al. suggested that IL-6-induced vascular endothelial growth factor expression and angiogenesis were inhibited through the JAK2/STAT3 pathway in rheumatoid arthritis, providing novel insights into antiangiogenic activity in rheumatoid arthritis (18). We concluded that when JAK2 inhibitors are used to treat myelofibrosis, they also inhibit IL-6-induced JAK2-related signal transduction in the lungs to achieve therapeutic effects. Compared with other treatments for ILDs such as hormones and immunosuppressants, the curative effect of ruxolitinib was more significant than that of other treatments, including prednisone, cyclophosphamide, anti-fibrotic agents, and gamma globulin. **Table 1** shows the results of the hemogram and inflammation index following treatment with ruxolitinib, which was consistent with our patient’s clinical manifestations.

We hypothesized that *TET2* mutation would lead to an increased IL-6 level, activating the JAK signaling pathway and leading to an intense inflammatory reaction. This may explain interstitial pneumonia in myelofibrosis and how treatment with *JAK2* inhibitors to block the IL-6-mediated *JAK2* downstream signaling pathway also blocked the final inflammatory reaction in the lung.

The *JAK2* inhibitor ruxolitinib, used to treat PMF, has been shown to be effective in alleviating symptoms, diminishing spleen size, and improving the survival rates of patients with *JAK2* mutations (19). However, the variability in patient responses highlights the necessity for personalized treatment plans and for the exploration of combination therapies to ensure optimal results.

## 4 Conclusion

PMF can involve different systems other than the hematological system, as in our case, which involved the respiratory system. Treatment with a *JAK2* inhibitor may act as a “two birds with one stone” approach to treat patients with PMF and ILD. A limitation of our case report is that only a single case is reported. Further case reports involving larger patient numbers are needed to validate our findings.

## Data Availability

The datasets presented in this article are not readily available because all data are in manuscript. Requests to access the datasets should be directed to xiaopp0026@163.com.
